# A Meta‐Analysis of Efficacy and Safety of Neoadjuvant Immunotherapy Plus Chemotherapy for Resectable Non‐Small Cell Lung Cancer

**DOI:** 10.1111/crj.70019

**Published:** 2024-10-02

**Authors:** Xinru Sun, Tianhua Kang, Baodong Liu, Yin Zhang, Guangming Huang

**Affiliations:** ^1^ Department of Interventional Oncology Zibo Central Hospital Zibo Shandong Province China

**Keywords:** chemotherapy, immunotherapy, neoadjuvant therapy, NSCLC

## Abstract

**Introduction:**

Neoadjuvant immunotherapy plus chemotherapy has ushered in a new era for surgical treatment for patients with NSCLC. This study aimed to examine the efficacy and safety of neoadjuvant immunotherapy plus chemotherapy in NSCLC.

**Methods:**

Eligible studies were identified from PubMed, Embase, Web of Science, Cochrane Library, ClinicalTrials.gov, and conference meeting abstracts. The endpoints included major pathological response (MPR), complete pathological response (pCR), surgical resection rate, R0 resection, treatment‐related adverse events (TRAEs), severe adverse events (SAEs), surgical complications, treatment discontinuation, surgical delay, and treatment‐related death. Stata 18 software was used for statistical analysis, and *p* < 0.05 was considered statistically significant. Twenty‐two studies including a total of 1108 patients were eligible for this study.

**Results:**

Among the patients who received neoadjuvant immunotherapy plus chemotherapy, the pooled MPR rate was 51% (95% CI [0.44–0.58]), and pCR rate was 34% (95% CI [0.28–0.40]). The pooled surgical resection rate was 85% (95% CI [0.81–0.89]), and the pooled R0 rate was 94% (95% CI [0.91–0.96]). The pooled rate of pathological tumor downstaging was 84% (95% CI [0.79–0.88]), and the pooled rate of pathological nodal downstaging was 38% (95% CI [0.23–0.57]). During the treatment of neoadjuvant immunotherapy plus chemotherapy with or without surgery, the pooled rate of TRAEs (any grade) was 84% (95% CI [0.73–0.91]), and the pooled rate of SAEs was 29% (95% CI [0.21–0.38]). Surgical complications pooled rate was 25% (95% CI [0.14–0.41]). The pooled rate of treatment discontinuation (11%, 95% CI [0.09–0.13]), surgical delay (3%, 95% CI [0.02–0.05]), and treatment‐related death (2%, 95% CI [0.02–0.03]) were conducted.

**Conclusion:**

Neoadjuvant immunotherapy plus chemotherapy provides a high pathological response, surgical resection rate, R0 resection rate, and pathological downstage rate and has a low risk of increasing the incidence of SAEs, surgical complications, treatment discontinuation, surgical delay, and treatment‐related death. The validation of prospective and large sample studies is needed to confirm this conclusion.

## Graphical abstract

1. Neoadjuvant immunotherapy plus chemotherapy is demonstrated to be effective and safe for patients with NSCLC. 2. Future research should evaluate the survival benefits of combining neoadjuvant immunotherapy with chemotherapy. 3. The adverse reactions associated with neoadjuvant immunochemotherapy exist in the whole course of treatment and should be treated with caution.

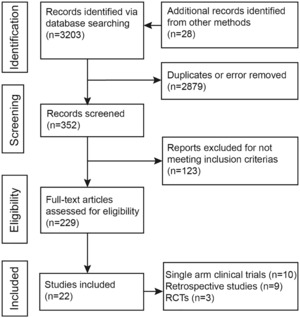

## 1 Introduction

Lung cancer is the leading cause of cancer death worldwide according to cancer statistics in 2022 [1]. Non‐small cell lung cancer (NSCLC) accounts for about 85% of all lung cancers [2]. Patients with NSCLC are diagnosed at an advanced stage in approximately 70%, with a 5‐year survival rate of less than 18% [3]. Surgical resection is the primary treatment for early‐stage NSCLC, which provides a high cure rate. However, patients suitable for complete surgical resection have a high risk of recurrence, ranging from 25% to 70% [4]. Neoadjuvant or adjuvant radiotherapy or chemotherapy can increase the 5‐year survival rate by only 5% [5, 6]. Thus, novel neoadjuvant regimens are urgently needed to decrease the risk of disease recurrence and increase the survival time of patients with NSCLC.

Immunotherapy has become an important component in the treatment of advanced NSCLC, with the potential to significantly improve 5‐year overall survival rates [7]. Chemotherapy has served as the cornerstone of treatment for advanced cancer for decades. Recent clinical trials have explored the combination of immunotherapy with immune checkpoint inhibitors (ICIs) and chemotherapy for NSCLC, with some showing notable improvements in the major pathological response (MPR) and complete pathological response (pCR) rates [3, 8]. Based on the clinical evidence, the Food and Drug Administration (FDA) approved the use of neoadjuvant nivolumab plus platinum‐doublet chemotherapy for NSCLC [9]. However, the European Medicines Agency granted approval for the application of nivolumab combined with platinum‐based chemotherapy only for NSCLC patients who are at high risk of recurrence and exhibit tumor cell PD‐L1 levels exceeding 1% [10]. Existing evidence has shown that neoadjuvant immunochemotherapy is superior to neoadjuvant monotherapy (immunotherapy or chemotherapy alone) in the treatment of resectable NSCLC. A meta‐analysis concluded that compared with neoadjuvant immunotherapy alone, neoadjuvant immunotherapy plus chemotherapy significantly improved the rate of MPR or pCR without increasing the occurrence of severe adverse events (SAEs) or surgical delay [3]. Consistently, another meta‐analysis confirmed that compared with neoadjuvant monotherapy (immunotherapy or chemotherapy alone), neoadjuvant immunotherapy plus chemotherapy achieved more pathological and radiological responses, and a lower risk of surgical complications and SAEs [11]. These studies of pathological reactions and adverse reactions caused by immunotherapy combined with or without chemotherapy have attracted extensive attention from clinicians.

To date, an increasing number of clinical trials and studies have been initiated to evaluate neoadjuvant immunotherapy combined with chemotherapy for the treatment of resectable NSCLC. However, there is a lack of comprehensive analysis on the incidence of pathological reactions and adverse reactions. Moreover, most studies reporting on neoadjuvant immunotherapy plus chemotherapy involve small sample sizes, which limits the strength of the evidence. To enhance the confidence level of these findings, pooling data from independent and similar clinical trials and observational studies can increase precision. Here, we conducted an updated meta‐analysis to evaluate the efficacy and safety of neoadjuvant immunotherapy plus chemotherapy in NSCLC, and offer a reference for treatment options.

## 2 Method

### 2.1 Search Strategy and Study Selection

We searched PubMed, Embase, Web of Science, Cochrane Library, ClinicalTrials.gov, and the meeting abstracts of conferences (ASCO, ESMO, and AACR) from January 1, 2020, to September 1, 2024, to identify eligible studies. The following terms were used for the search query: (neoadjuvant) AND (Chemotherapy OR immunotherapy OR immunochemotherapy OR chemoimmunotherapy) AND (non‐small cell lung cancer OR lung cancer OR pulmonary carcinoma).

### 2.2 Inclusion and Exclusion Criteria

Inclusion criteria were listed as follows: (a) patients: patients diagnosed with resectable NSCLC; (b) intervention: neoadjuvant chemoimmunotherapy before surgery; (c) comparison: no restriction on whether control groups or intervention measures were set up; (d) outcomes: the endpoints such as MPR rate for surgical patients, pCR rate for surgical patients, surgical resection rate, R0 resection rate, tumor downstaging rate, nodal downstaging rate, and adverse reactions including TRAEs, SAEs, surgical complications, treatment discontinuation, surgical delay, and treatment‐related death; (e) Study design: randomized controlled trials (RCTs), non‐RCTs, single‐arm clinical studies and trials. Exclusion criteria were as follows: (a) treatment with monotherapy or other combination therapies other than chemotherapy plus immunotherapy; (b) studies that did not report the required data on efficacy and safety; (c) studies that were not available; (d) duplicate studies.

### 2.3 Data Extraction

We extracted the data and gathered the following information: (a) characteristics of studies and patients including first author's name, sample size, published years, basic study design, National Clinical Trials (NCT) registry number, and study phase, neoadjuvant treatment regimens, median age, proportion of males and squamous‐cell carcinoma; (b) endpoints: MPR, pCR, surgical resection rate, R0 resection rate, tumor downstaging rate, nodal downstaging rate, incidence of any grade TRAEs, SAEs, surgical complications, treatment discontinuation, surgical delay, and treatment‐related death.

### 2.4 Statistical Analysis

All meta‐analyses were performed with single rate meta‐analysis because most of the included studies were single‐arm clinical trials and the random‐effects model (DerSimonian–Laird method) was adopted. The effect size was all the pooled prevalence proportions with 95% confidence intervals (CIs). The heterogeneity was evaluated by *I* ^2^ test and the Cochran *Q* test: *I* ^2^ < 50% was considered as negligible heterogeneity, *I* ^2^ ranging from 50% to 75% as moderate heterogeneity and *I* ^2^ ≥ 75% as substantial heterogeneity, respectively [12], or *p* < 0.05 indicated the presence of significant heterogeneity. The non‐parametric trim‐and‐fill analysis was performed to evaluate publication bias. Stata18 software was used for this study. *p* < 0.05 was considered statistically significant.

## 3 Results

### 3.1 The Characteristics of Studies and Patients

**FIGURE 1:**
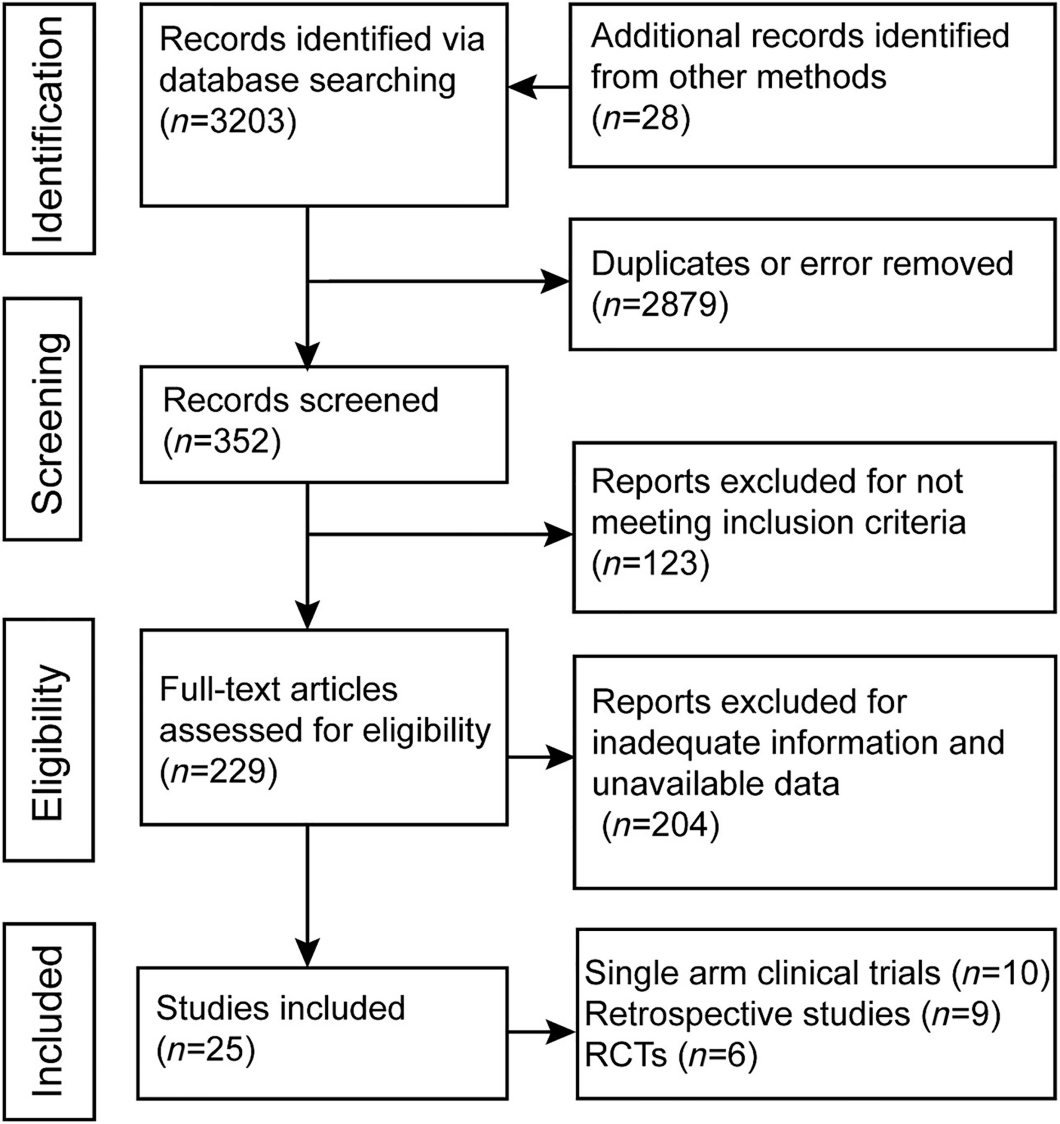
Flowchart of study selection.

A total of 25 studies met the inclusion criteria and 2069 patients were finally enrolled. Figure 1 depicts the flowchart of study selection procedure. There were 10 single arm clinical trials [13, 14, 15, 16, 17, 18, 19, 20, 21, 22], 9 retrospective studies [23, 24, 25, 26, 27, 28, 29, 30, 31], and 6 RCT studies [32, 33, 34, 35, 36, 37]. All the retrospective studies were single‐arm studies. Among the included 2069 patients, the median age was 63 years old, the proportion of males was 18%–96%, and squamous cell carcinoma ranged from 33% to 92%. The summary of included studies and patients was shown in Table 1.

**TABLE 1:** Studies and patients characteristics.

First author	Year	Study design	NCT number	Study phase	Neoadjuvant treatment regimen	Neoadjuvant cycle	Stage	Sample size	Median age (years)	Proportion of male (%)	Proportion of SCC (%)
Sun	2023	Single‐center, single‐arm	NCT04326153	2	Sintilimab + nab‐paclitaxel + carboplatin	2–3 circles	IIIA/IIIB	20	59.5 (34–71)	90% (18/20)	80% (16/20)
Zhang	2022	Single‐center, single‐arm	NR	2	Sintilimab + carboplatin +gemcitabine	2–4 circles	IB‐IIIA	50	64.84 ± 9.61	88% (44/50)	56% (28/50)
Hou	2022	Single‐center, single‐arm	NR	1	Toripalimab + platinum‐paclitaxel	2–4 circles	II‐IIIB	11	63 (51–71)	90.9% (10/11)	81.8% (9/11)
Zhu	2022	Single‐center, single‐arm	NR	2	Toripalimab + carboplatin	2–4 circles	II‐III	50	66 (57.8–68.0)	84% (42/50)	64.0% (32/50)
Yan	2022	Single‐center, single‐arm	NCT04606303	2	Toripalimab + cisplatin‐based chemotherapy	2–4 circles	IIB‐IIIB	53	62 (45–76)	91% (48/53)	79% (42/53)
Zhao	2021	Single‐center, single‐arm	NCT04304248	2	Toripalimab +carboplatin +pemetrexed/nab‐paclitaxel	3 circles	IIIA or IIIB	33	61 (56–66)	18% (6/33)	55% (18/33)
Rothschild	2021	Multicentre, single‐arm	NCT02572843	2	Durvalumab + cisplatin + docetaxel	2–3 circles	IIIA	67	61 (41–74)	52% (35/67)	33% (22/67)
Duan	2021	Multicentre, single‐arm	NR	NR	Chemoimmunotherapy	3–4 cycles	IIA–IIIB	23	61.83	96% (22/23)	83% (19/23)
Shu	2020	Multicentre, single‐arm	NCT02716038	2	Atezolizumab + nab‐paclitaxel + carboplatin	2–4 circles	IB‐IIIA	30	67 (62–74)	50% (15/30)	40% (12/30)
Provencio	2020	Multicentre, single‐arm	NCT03081689	2	Nivolumab + paclitaxel + carboplatin	3 circles	IIIA	46	63 (58–70)	74% (34/46)	35% (16/46)
Fang	2023	Retrospective study	NR	NR	PD‐1 inhibitors + platinum	2 circles	II‐III	211	64 (38–77)	92.9% (196/211)	82% (172/211)
Chen	2022	Retrospective study	NR	NR	Pembrolizumab/Nivolumab+ carboplatin + paclitaxel	2–4 cycles	IIIA/IIIB	12	61(55.25–66.75)	75% (9/12)	33% (4/12)
Hong	2021	Retrospective study	NR	NR	Neoadjuvant chemoimmunotherapy	2–4 cycles	IIA‐IIIC	25	62 (51–83)	92% (23/24)	76% (19/25)
Wang	2021	Retrospective study	NR	NR	PD‐1 inhibitors + albumin paclitaxel +carboplatin	2 circles	IIIA	72	62.2 (42–76)	92% (66/72)	92% (66/72)
Zhai	2021	Retrospective study	NR	NR	Nivolumab + paclitaxel + carboplatin	3 circles	IIIA/IIIB	46	63 (56–73)	56.5% (26/46)	58.7% (27/46)
Chen	2021	Retrospective study	NR	NR	Pembrolizumab plus chemotherapy	2 circles	IIIA/IIIB	35	62.17 ± 5.99	83% (29/35)	75% (26/35)
Shen	2021	Retrospective study	NR	NR	Pembrolizumab + albumin‐bound paclitaxel + carboplatin	2 circles	IIB‐IIIB	37	62.8(38–76)	94.6% (35/37)	100% (37/37)
Tfayli	2020	Retrospective study	NR	NR	Avelumab + neoadjuvant chemotherapy	4 cycles	IB/II/IIIA	15	65 (45–80)	46.7% (7/15)	13.3% (2/15)
Dai	2022	Retrospective study	NR	NR	Neoadjuvant chemoimmunotherapy	2–4 cycles	IB/IIIA/IIIB	23	63.2 ± 7.0	96% (22/23)	78% (18/23)
Liu	2023	Multicentre, randomized	NCT04015778	2	Nivolumab + nab‐paclitaxel + carboplatin	3 circles	IIA–IIIB	12	64 (43–78)	83.3% (10/12)	41.7% (5/12)
Forde	2022	Multicentre, randomized	NCT02998528	3	Nivolumab + platinum‐based chemotherapy	3 circles	IB‐IIIA	176	64 (41–82)	71.5% (128/179)	48.6% (87/179)
Provencio	2022	Multicentre, randomized	NCT03838159	2	Nivolumab + paclitaxel + carboplatin	3 circles	IIIA	57	65 (58–70)	63% (36/57)	37% (21/57)
Wakelee	2023	Multicentre, randomized	NCT03425643	3	Pembrolizumab + cisplatin‐based chemotherapy	4 circles	II, IIIA, or IIIB (N2)	397	63 (26–83)	70.3% (279/397)	43.1% (171/397)
Heymach	2023	Multicentre, randomized	NCT03800134	3	Durvalumab + platinum‐based chemotherapy	4 circles	II to IIIB (N2)	366	65 (30–88)	68.9% (252/366)	46.2% (169/366)
Lu	2024	Multicentre, randomized	NCT04158440		Toripalimab + platinum‐based chemotherapy	3 circles	II, IIIA, or IIIB (N2)	202	62 (56–65)	89.6% (181/202)	77.7% (157/202)

### 3.2 The Efficacy of Neoadjuvant Immunotherapy Plus Chemotherapy

**FIGURE 2:**
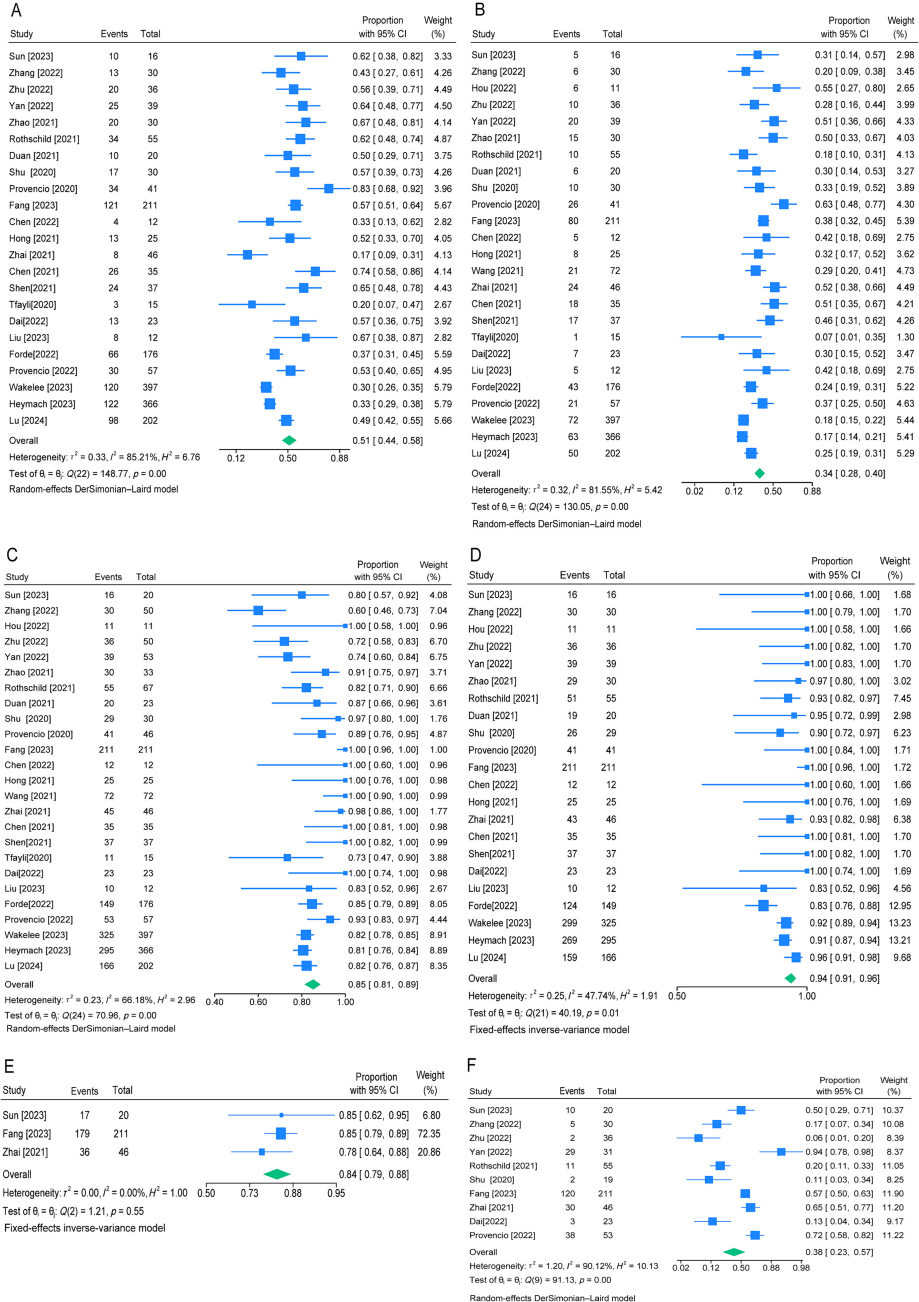
Forest plots of the efficacy of neoadjuvant immunotherapy plus chemotherapy. (A) The pooled MPR rate. (B) The pooled pCR rate. (C) The pooled rate of surgical resection rate. (D) The pooled rate of R0 resection. (E) The pooled rate of pathological tumor downstaging. (F) The pooled rate of pathological nodal downstaging.

The efficacy was summarized in Table 2, and the pathological response outcomes were shown in forest plots in Figure 2. Twenty‐five studies reported the rate of MPR. Among the 1911 patients enrolled, 839 achieved MPR and generated a pooled prevalence of 51% (95% CI [0.44–0.58], *I* ^2^ = 85%, *p* < 0.01) (Figure 2A). Five hundred forty‐nine patients achieved pCR among the 1994 patients enrolled and the pooled pCR rate was 34% (95% CI [0.28–0.40], *I* ^2^ = 82%, *p* < 0.01) (Figure 2B). A total of 2069 patients were enrolled, of which 1776 underwent surgery. The pooled prevalence of surgical resection rate was 85% (95% CI [0.81–0.89], *I* ^2^ = 66%, *p* < 0.01) (Figure 2C), ranging from 72% to 100%. R0 resection represents no residual tumor. The occurrence of R0 resection varied from 83% to 100%, with pooled rate of 94% (95% CI [0.91–0.96], *I* ^2^ = 48%, *p* = 0.01) (Figure 2D). The pooled incidence of pathological tumor downstage was 84% (95% CI [0.79–0.88], *I* ^2^ = 0%, *p* = 0.55) (Figure 2E), and pathological nodal downstage was 38% (95% CI [0.23–0.57], *I* ^2^ = 90%, *p* < 0.01) (Figure 2F).

**TABLE 2:** The efficacy of neoadjuvant chemoimmunotherapy.

First author	Patients with resection	R0 resection rate	MPR rate	pCR rate	Pathological tumor downstage	Pathological nodal downstage
Sun	80% (16/20)	100% (16/16)	63% (10/16)	31% (5/16)	85% (17/20)	50% (10/20)
Zhang	60% (30/50)	100% (30/30)	43% (13/30)	20% (6/30)	NR	17% (5/30)
Hou	100% (11/11)	100% (11/11)	NR	55% (6/11)	NR	NR
Zhu	72% (36/50)	100% (36/36)	56% (20/36)	28% (10/36)	NR	6% (2/36)
Yan	74% (39/53)	100% (39/39)	64% (25/39)	51% (20/39)	NR	94% (29/31)
Zhao	91% (30/33)	97% (29/30)	67% (20/30)	50% (15/30)	NR	NR
Rothschild	82% (55/67)	93% (51/55)	62% (34/55)	18% (10/55)	NR	20% (11/55)
Duan	87% (20/23)	95% (19/20)	50% (10/20)	30% (6/20)	NR	NR
Shu	97% (29/30)	90% (26/29)	57% (17/30)	33% (10/30)	NR	11% (2/19)
Provencio	89% (41/46)	100% (41/41)	83% (34/41)	63% (26/41)	NR	NR
Fang	100% (211/211)	100% (211/211)	57% (121/211)	38% (80/211)	85% (179/211)	57% (120/211)
Chen	100% (12/12)	100% (12/12)	33% (4/12)	42% (5/12)	NR	NR
Hong	100% (25/25)	100% (25/25)	52% (13/25)	32% (8/25)	NR	NR
Wang	100% (72/72)	NR	NR	29% (21/72)	NR	NR
Zhai	98% (45/46)	96% (43/46)	17% (8/46)	52% (24/46)	78% (36/46)	65% (30/46)
Chen	100% (35/35)	100% (35/35)	75% (26/35)	51% (18/35)	NR	NR
Shen	100% (37/37)	100% (37/37)	65% (24/37)	46% (17/37)	NR	NR
Tfayli	73% (11/15)	NR	20% (3/15)	7% (1/15)	NR	NR
Dai	100% (23/23)	100% (23/23)	57% (13/23)	27% (7/23)	NR	13% (3/23)
Liu	83% (10/12)	100% (10/10)	67% (8/12)	42% (5/12)	NR	NR
Forde	83% (149/176)	83% (124/149)	37% (66/176)	24% (43/176)	NR	NR
Provencio	93% (53/57)	NR	53% (30/57)	37% (21/57)	NR	72% (38/53)
Wakelee	82% (325/397)	92% (299/325)	30% (120/397)	18% (72/397)	NR	NR
Heymach	81% (295/366)	91% (269/295)	33% (122/366)	17% (63/366)	NR	NR
Lu	82% (166/202)	96% (159/166)	49% (98/202)	25% (50/202)	NR	NR

### 3.3 The Safety of Neoadjuvant Immunotherapy Plus Chemotherapy

**FIGURE 3:**
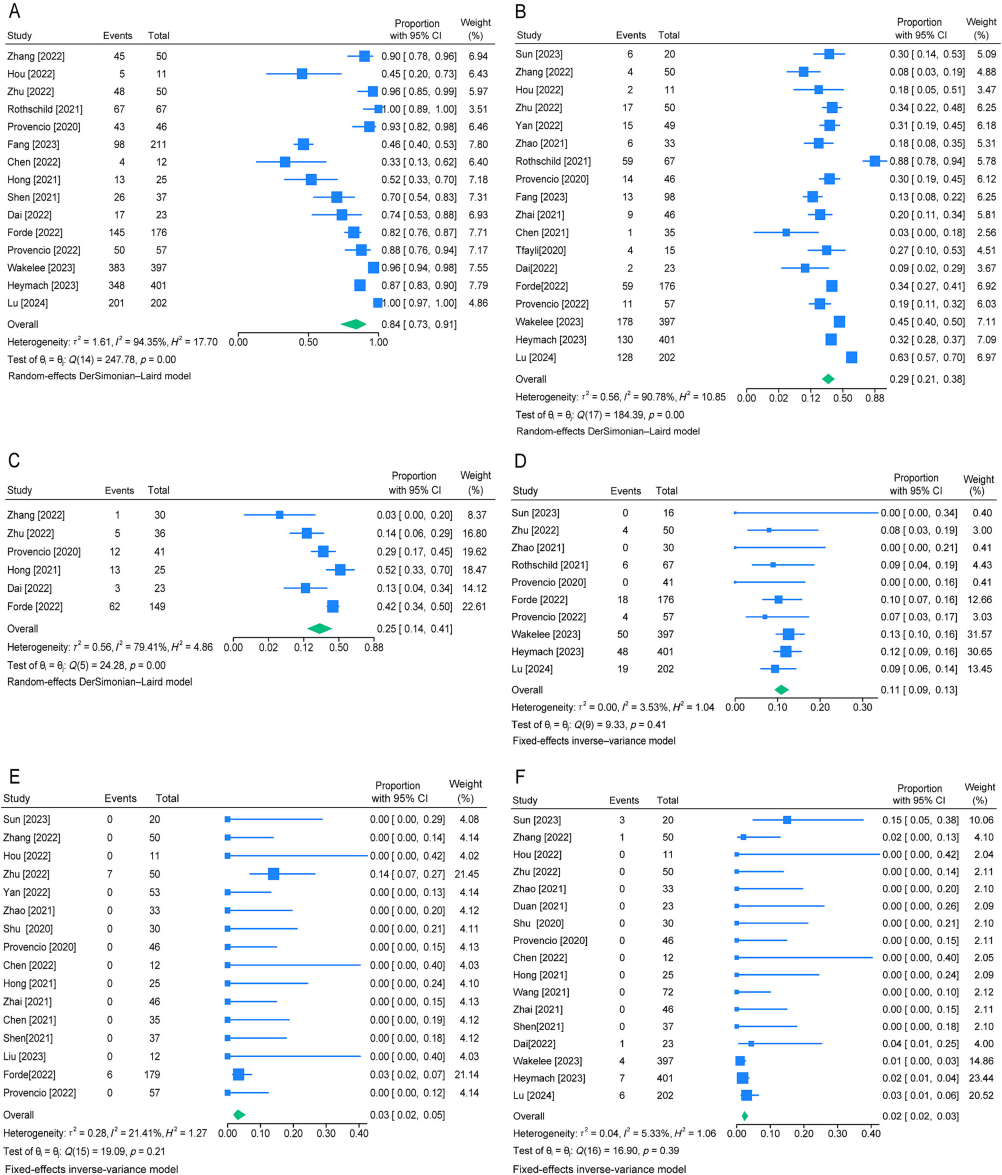
Forest plots of the safety of neoadjuvant immunotherapy plus chemotherapy. (A) The pooled any grade TRAEs rate. (B) The pooled rate of SAEs. (C) The pooled rate of surgical complications. (D) The pooled incidence of treatment discontinuation. (E) The pooled incidence of surgical delay; F. The pooled incidence of treatment/surgery related death.

The safety of neoadjuvant chemotherapy plus immunotherapy was summarized in Table 3 and forest plots were presented in Figure 3. Treatment‐related adverse events (TRAEs) and severe adverse events (SAEs) were based on the Common Terminology Criteria for Adverse Events (CTCAE). SAEs were defined as grade 3–5 TRAEs. Fifteen studies provided the incidence of any grade TRAEs, and the pooled TRAEs rate was 84% (95% CI [0.73–0.91], *I* ^2^ = 94%, *p* < 0.01), ranging from 33% to 100% (Figure 3A). Eighteen studies reported the incidence of SAEs and the pooled rate of SAEs ranged from 13% to 88% with a pooled incidence of 29% (95% CI [0.21–0.38], *I* ^2^ = 91%, *p* < 0.01) (Figure 3B). The pooled rate of surgical complications was 25% (95% CI [0.14–0.41], *I* ^2^ = 79%, *p* < 0.01) (Figure 3C). Treatment discontinuation was observed in 149 of 1437 patients; the pooled incidence was 11% (95% CI [0.09–0.13], *I* ^2^ = 4%, *p* = 0.41), ranging from 0% to 13% (Figure 3D). The pooled rate of surgical delay was 3% (95% CI [0.02–0.05], *I* ^2^ = 21%, *p* = 0.21) (Figure 3E), and the treatment or surgical death pooled rate was 2% (95% CI [0.02–0.03], *I* ^2^ = 5%, *p* = 0.39) (Figure 3F).

**TABLE 3:** The safety of neoadjuvant chemoimmunotherapy.

First author	Incidence of any grade TRAEs	Incidence of SAEs	Incidence of surgical complications	Treatment discontinuation	Surgical delay	Treatment/surgical‐related death
Sun	NR	30% (6/20)	NR	0	0	15% (3/20)
Zhang	90% (45/50)	8% (4/50)	3% (1/30)	NR	0	2% (1/50)
Hou	45% (5/11)	18% (2/11)	NR	NR	0	0
Zhu	96% (48/50)	34% (17/50)	14% (5/36)	8% (4/50)	14% (7/50)	0
Yan	NR	31% (15/49)	NR	NR	0	NR
Zhao	NR	18% (6/33)	NR	0	0	0
Rothschild	100% (67/67)	88% (59/67)	NR	9% (6/67)	NR	NR
Duan	NR	NR	NR	NR	NR	0
Shu	NR	NR	NR	NR	0	0
Provencio	93% (43/46)	30% (14/46)	29% (12/41)	0	0	0
Fang	46.4% (98/211)	13% (13/98)	NR	NR	NR	NR
Chen	33% (4/12)	NR	NR	NR	0	0
Hong	52% (13/25)	NR	52% (13/25)	NR	0	0
Wang	NR	NR	NR	NR	NR	0
Zhai	NR	20% (9/46)	NR	NR	0	0
Chen	NR	3% (1/35)	NR	NR	0	NR
Shen	70% (26/37)	NR	NR	NR	0	0
Tfayli	NR	27% (4/15)	NR	NR	NR	NR
Dai	74% (17/23)	9% (2/23)	13% (3/23)	NR	NR	4% (1/23)
Liu	NR	NR	NR	NR	0	NR
Forde	82% (145/176)	34% (59/176)	42% (62/149)	10% (18/176)	3% (6/176)	0
Provencio	88% (50/57)	19% (11/57)	NR	7% (4/57)	0	NR
Wakelee	97% (383/397)	45% (178/397)	NR	13% (50/397)	NR	1% (4/397)
Heymach	87% (348/401)	32% (130/401)	NR	12% (48/401)	NR	1.7% (7/401)
Lu	99.5(201/202)	63% (128/202)	NR	9% (19/202)	NR	3% (6/202)

### 3.4 Publication Bias

**FIGURE 4:**
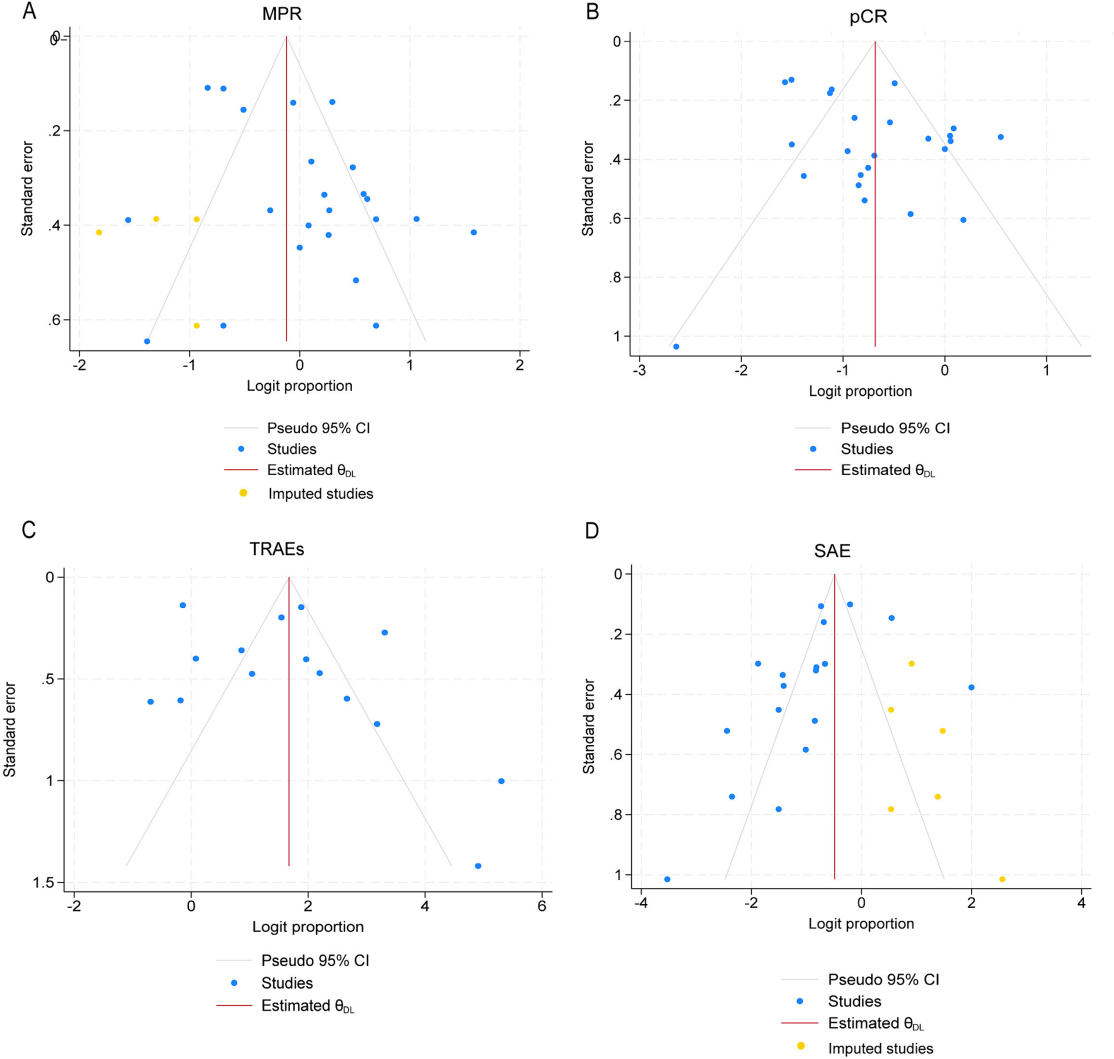
Funnel plots of MPR, pCR, TRAEs and SAEs in patients with NSCLC undergoing neoadjuvant immunotherapy plus chemotherapy.

Funnel plots of MPR, pCR, TRAEs, and SAEs were shown in Figure 4. Due to the high heterogeneity, the random effect model was used in this study. Large‐scale RCTs are still needed to be performed to evaluate the neoadjuvant regimens on the prognosis of NSCLC. The funnel plots were presented and non‐parametric trim‐and‐fill analysis showed that *p* > 0.05, with no obvious publication bias.

## 4 Discussion

Neoadjuvant immunochemotherapy is emerging as a viable treatment option for patients with NSCLC. The rationale for this combination is that immunotherapy can target and eliminate disseminated and metastatic tumor but is less effective against solid tumor masses [38]. Chemotherapy can improve the effectiveness of immunotherapy by shrinking the primary tumor, reducing the number of cells that immune cells need to eliminate, and lowering the immunosuppressive factors produced by tumor cells. Moreover, some chemotherapeutic agents can directly activate antitumor immunity, which is particularly significant for ‘cold’ tumors with low effector T cell infiltration [38]. Based on this, multiple clinical studies and trials have explored the effectiveness and safety of neoadjuvant ICIs and chemotherapeutics using various treatment regimens in NSCLC. CheckMate 816 trial [32] demonstrated that neoadjuvant ICIs plus chemotherapy showed a significantly higher MPR rate (37% vs. 9%) and pCR rate (24% vs. 2%) than chemotherapy alone. The incidence of TRAEs of any grade was 92.6% in the ICIs plus chemotherapy group versus 97.2% in the chemotherapy‐alone group, while SAEs occurred in 33.5% and 36.9% of patients, respectively. Surgical complications were observed in 41.6% of the ICIs plus chemotherapy group and 46.7% of the chemotherapy‐alone group. The NEOSTAR trial [39] demonstrated significant findings in the treatment of resectable NSCLC using combination immunotherapy. Specifically, the combination of ipilimumab and nivolumab resulted in twice as many MPRs as nivolumab alone, with rates of 50% compared with 24%. Furthermore, the combination therapy achieved a greater proportion of pCRs, at 38% versus 10% for nivolumab alone. In terms of safety, SAEs were reported in 13% (3 out of 23) of patients treated with nivolumab and 10% (2 out of 21) of those receiving the combination of nivolumab and ipilimumab.

In this study, our results indicated a pooled MPR rate of 51%, which seemed higher than the previously published data on immunochemotherapy and dual ICIs; while the pooled pCR rate was 34%, which was highly variable across different studies. Additionally, our study revealed that the pooled surgical resection rate was 88% and R0 resection rate was 91%. Pathological tumor downstage pooled rate was 84% and pathological nodal downstage pooled rate was 38%. These results underscore the potential benefits of immunochemotherapy for NSCLC with high pathological responses and good surgical outcomes. Regarding safety, the pooled incidence of TRAEs of any grade was 84% and SAEs was 29%. The pooled rates of surgical complications, surgical delay, treatment discontinuation, and treatment‐related death were 25%, 3%, 11%, and 2%, respectively. However, although the combination approach rarely develops life‐threatening adverse events, this combination also raises concerns about TRAEs, particularly in patients with poor clinical performance status [40, 41]. For these patients, there is a need for a comprehensive assessment of individual benefits and risks when selecting neoadjuvant therapy regimens in clinical practice.

This study has several limitations. First, although there are 25 studies included in this meta‐analysis, the sample sizes of studies are small, and the majority of them are non‐randomized single‐arm trials. As a result, it is difficult to conduct comparative analyses and derive statistically significant conclusions. Second, due to the short follow‐up periods of the included studies, data reporting on survival outcomes is not complete. According to the available data from NADIM II trial [33], neoadjuvant immunochemotherapy has shown significant improvements in progression‐free survival (PFS) and overall survival (OS) compared with neoadjuvant chemotherapy alone. At 24 months, PFS was 67.2% for immunochemotherapy versus 40.9% for chemotherapy (HR: 0.47, 95% CI [0.25–0.88]), and OS was 85.0% compared with 63.6% (HR: 0.43, 95% CI [0.19 to 0.98]). In alignment with these results, a recent meta‐analysis showed superior event‐free survival (HR: 0.59, 95% CI [0.52–0.67]) and OS (HR: 0.65, 95% CI [0.54–0.79]) with neoadjuvant immunochemotherapy over neoadjuvant chemotherapy [42]. Third, ICIs (including sintilimab, toripalimab, durvalumab, nivolumab, pembrolizumab, and atezolizumab) and chemotherapeutics (paclitaxel, carboplatin, gemcitabine, and platinum) vary among different studies. This diversity may contribute to the significant heterogeneity across studies and complicate the ability to perform subgroup analyses. Given these challenges, there is a clear need for future research involving large‐scale, multicenter, and well‐designed studies with long follow‐up periods. Nonetheless, by synthesizing data from multiple trials, our study sought to provide a comprehensive overview of the treatment landscape, potentially offering a useful reference for both clinicians and researchers. With an increasing number of active clinical trials on immunochemotherapy, we look forward to more definitive evidence regarding the effectiveness and safety of immunochemotherapy, helping to standardize treatment protocols and improve patient outcomes in NSCLC.

## Conclusion

This meta‐analysis confirmed the effectiveness of immunochemotherapy for treating NSCLC given that it was associated with high pathological responses, surgical resection rates, and pathological tumor downstaging rates. In terms of safety, although immunochemotherapy presents a low risk of SAEs, surgical complications, surgical delay, treatment discontinuation, and death, some common treatment‐related adverse reactions associated with this combination therapy exist throughout the course of treatment, which should be treated with caution. Future research should evaluate the survival benefits of immunochemotherapy in patients with NSCLC. Moreover, it is crucial to continue to investigate the optimal dosage, timing, specific type, and sequence of these treatment regimens.

## Author Contributions

**Xinru Sun:** conceptualization, data curation, methodology, software, writing – original draft. **Tianhua Kang:** formal analysis, project administration, visualization. **Baodong Liu:** formal analysis, project administration, visualization. **Yin Zhang:** formal analysis, project administration, visualization. **Guangming Huang:** investigation, supervision, validation, writing – review and editing.

## Ethics Statement

The authors have nothing to report.

## Conflicts of Interest

The authors declare no conflict of interest.

## Data Availability

The data are available from the corresponding author upon reasonable request.
